# *JAGGED* Controls Growth Anisotropy and Coordination between Cell Size and Cell Cycle during Plant Organogenesis

**DOI:** 10.1016/j.cub.2012.07.020

**Published:** 2012-10-09

**Authors:** Katharina Schiessl, Swathi Kausika, Paul Southam, Max Bush, Robert Sablowski

**Affiliations:** 1 Department of Cell and Developmental Biology, John Innes Centre, Norwich Research Park, Norwich NR4 7UH, UK; 2 School of Computing Sciences, University of East Anglia, Norwich Research Park, Norwich NR4 7TJ, UK

## Abstract

**Background:**

In all multicellular organisms, the links between patterning genes, cell growth, cell cycle, cell size homeostasis, and organ growth are poorly understood, partly due to the difficulty of dynamic, 3D analysis of cell behavior in growing organs. A crucial step in plant organogenesis is the emergence of organ primordia from the apical meristems. Here, we combined quantitative, 3D analysis of cell geometry and DNA synthesis to study the role of the transcription factor JAGGED (JAG), which functions at the interface between patterning and primordium growth in *Arabidopsis* flowers.

**Results:**

The floral meristem showed isotropic growth and tight coordination between cell volume and DNA synthesis. Sepal primordia had accelerated cell division, cell enlargement, anisotropic growth, and decoupling of DNA synthesis from cell volume, with a concomitant increase in cell size heterogeneity. All these changes in growth parameters required *JAG* and were genetically separable from primordium emergence. Ectopic JAG activity in the meristem promoted entry into S phase at inappropriately small cell volumes, suggesting that JAG can override a cell size checkpoint that operates in the meristem. Consistent with a role in the transition from meristem to primordium identity, JAG directly repressed the meristem regulatory genes *BREVIPEDICELLUS* and *BELL 1* in developing flowers.

**Conclusions:**

We define the cellular basis for the transition from meristem to organ identity and identify *JAG* as a key regulator of this transition. *JAG* promotes anisotropic growth and is required for changes in cell size homeostasis associated with accelerated growth and the onset of differentiation in organ primordia.

## Graphical abstract

**Graphical Abstract:**

**Figure:**
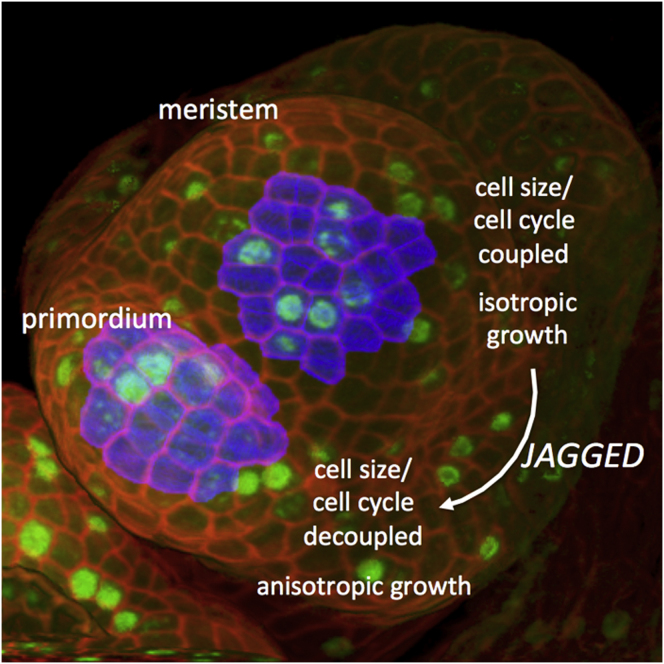

**Highlights:**

► *JAGGED (JAG*) is required for growth of initiating floral organs in *Arabidopsis* ► *JAG* decouples cell cycle from cell growth during organ emergence ► *JAG* promotes fast, anisotropic growth when floral organs emerge from the meristem ► *JAG* directly represses meristem identity genes

## Introduction

A fundamental question in biology is how the activity of regulatory genes acting within cells is translated into the shape and size of macroscopic organs. In plants, growth is based only on increased cell number and cell size, in contrast to animals, in which cell migration and cell death also play important roles. In spite of this simplifying feature, understanding the link between regulatory genes and the growth and shape of plant organs is still a considerable challenge. In theory, a complete understanding of growth would require information about rates, anisotropy, and directions of growth, and how these vary spatially and over time [1]. So far, these parameters have not been associated experimentally to specific regulatory genes, partly because of the difficulty of obtaining quantitative, dynamic, and three-dimensional (3D) information about organ growth. This situation has begun to change with new methods to analyze and model the dynamics of plant tissue growth in 3D [2, 3].

Shoot organs are initiated at the periphery of apical meristems, which continuously produce new cells to replenish those recruited into organ primordia [4]. These primordia develop into leaves during vegetative growth and into floral buds during the reproductive phase of development. Each bud contains its own floral meristem, which produces floral organ primordia in concentric whorls, with sepal primordia emerging first, followed by petal, stamen, and carpel primordia, after which the floral meristem is terminated. The spatial pattern of primordium initiation around the meristem (phyllotaxis) results from regulated transport of the phytohormone auxin [5]. Local auxin maxima induce primordium initiation, associated with repression of regulatory genes that maintain meristem activity, such as the homeodomain proteins SHOOT MERISTEMLESS (STM) [6] and BREVIPEDICELLUS (BP) [7, 8].

After primordia have been initiated, the growth of shoot organs is conventionally divided in two phases: primary morphogenesis, based mostly on cell proliferation, and secondary morphogenesis, based mostly on endoreduplication and cell expansion [9, 10]. Although multiple genes have been identified that control the final size and shape of plant organs, it remains largely unknown how these genes coordinate cell proliferation and expansion to determine organ shape and size [9, 11]. One of the regulators of the proliferative phase of organ growth in *Arabidopsis* is *JAGGED* (*JAG*), which encodes a zinc finger transcription factor expressed during the emergence of all shoot organs, but not in the meristem [12, 13]. In leaves, *jag* mutations cause serrated margins, and in floral organs cause reduced growth preferentially in the distal region. Based on reduced expression of histone H4 in *jag-1* petals, *JAG* function has been linked to cell proliferation during organ growth [12]. *JAG* is a direct target of the floral homeotic genes *AGAMOUS* and *SEPALATA3*, which are key patterning genes during floral bud development [14, 15]. Therefore, *JAG* appears to function at the interface between patterning genes and organ growth.

To understand the changes in cell behavior that mediate the effects of *JAG* on organ growth, we used recently developed methods for dynamic analysis of cell geometry and established a protocol for combined 3D analysis of cell geometry and patterns of DNA synthesis. Our analysis shows that cells in the meristem and early floral organ primordia have different growth regimes, and that *JAG* is a key regulator of the transition between these growth regimes. One of the unanticipated functions of *JAG* was to change the coordination between cell growth and cell cycle during organ development.

## Results

### *JAG* Promotes Changes in Growth Dynamics during the Transition from Meristem to Primordium Identity

Previous work on *jag* mutants focused on macroscopic phenotypes in leaves and floral organs at relatively late stages of development; defects in early organogenesis have been observed but have not been characterized in detail [12, 13]. We focused our analysis on the growth of sepal primordia, which are the first to emerge from the floral meristem and are therefore readily accessible for imaging.

To select buds at equivalent developmental stages in the wild-type (WT) and *jag-1*, we relied on the phyllotactic position of the buds around the inflorescence meristem. The spiral pattern of bud emergence was unaltered in *jag-1* compared to the WT (see Figure S1 available online); based on the rate of production of mature flowers in *jag-1* and in the WT, the frequency of bud initiation was also unchanged (data not shown). The size of the floral meristem was comparable in WT and *jag-1* buds at the same position, whereas growth of the sepal primordia was clearly inhibited in the mutant buds (Figure S1). Therefore the *jag-1* mutation specifically affects growth of the sepal primordia in early floral buds.

**Figure 1:**
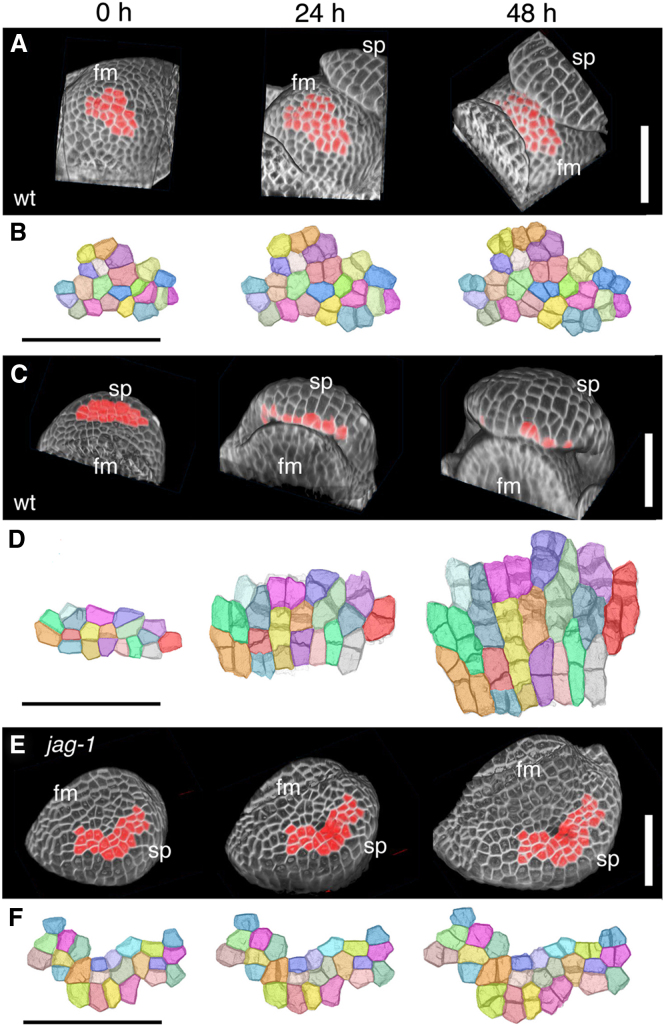
Live Imaging and Cell Tracking in WT and *jag-1* Buds fm, floral meristem; sp, sepal primordium; scale bars represent 50 μm. (A, C, and E) MARS 3D reconstructions of WT meristem (A), WT sepal primordium (C), and *jag-1* sepal primordium (E) of representative buds initially at phyllotactic positions 10–11 (0 hr), then imaged at 24 hr intervals; areas in red correspond to the groups of epidermal cells (sectors) shown in (B), (D), (F); in (C), most of the selected epidermal cells are not visible on the bud surface at 24 and 48 hr because the adaxial epidermis is hidden as the developing sepal curls over the floral meristem. (B, D, and F) Clonal analysis within the groups of cells colored red in (A), (C), (E); these sectors were virtually dissected from the bud and displayed as projections of cell wall images; each cell at 0 hr and its descendants at 24 and 48 hr (clone) are displayed in the same color; note the strongly oriented, anisotropic growth of the WT sepal epidermis (D), in contrast to the isotropic growth seen in the meristem (B) and *jag-1* sepal epidermis (F).

**Figure 2:**
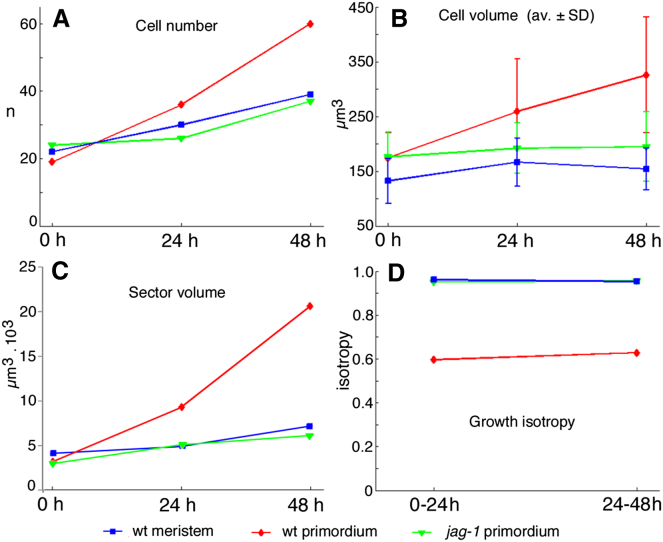
The *jag-1* Mutation Reverts the Growth Parameters of Sepal Primordia to Those of the Floral Meristem Growth parameters were quantified for each of the epidermal sectors shown in Figure 1; blue (WT meristem), red (WT primordium), and green (*jag-1* primordium) lines relate to the cells shown in Figures 1B, 1D, and 1F, respectively. (A) Increase in the number of cells per sector. (B) Average and SD of cell volume in each sector. (C) Total sector volume. (D) Isotropy of growth in each sector during each 24 hr interval. A value of 1 indicates similar growth in different directions, whereas values tending toward 0 indicate increasingly oriented growth.

To understand the cellular basis for the growth defects in *jag-1* buds, we compared the growth dynamics of the meristem and sepal primordia in the WT and in *jag-1*, using the 3D live imaging approach MARS (multiangle image acquisition, 3D reconstruction and cell segmentation) [2]. Sepal primordia started emerging in buds at position 9–10 in the phyllotactic spiral, so these were selected and imaged at 24 hr intervals over 2 days (Figures 1A, 1C, and 1E). We digitally marked groups of epidermal cells (sectors) in the initial bud and manually tracked the descendants of each cell (clone) over time (Figures 1B, 1D, and 1F). Within each virtual sector, we analyzed changes in cell number, cell volumes, total sector volume, and growth isotropy (i.e., the ratio between growth along the minimal and maximal growth axes) (Figure 2). We only considered isotropy of growth parallel to the epidermal surface, because the thickness of the epidermal cells remained uniform (data not shown).

As expected from the *JAG* expression pattern [12, 13] (Figure S1), growth of the floral meristem was comparable in the WT and in *jag-1*, so we only present the analysis of the WT meristem. Growth of sectors within the floral meristem was isotropic and sector growth was due primarily to increased cell numbers, whereas cell volumes and heterogeneity in cell and clone volumes remained approximately constant (Figures 1A, 1B, and 2). In contrast, sectors in the adaxial epidermis of the WT sepal primordium showed anisotropic growth, with maximum growth along the apical-basal axis; virtual sectors grew faster than in the meristem due to a combination of increased cell proliferation and increased cell volumes; the sepal primordium also showed increasing heterogeneity in cell volumes (Figures 1C, 1D, and 2). Strikingly, growth in the adaxial epidermis of the *jag-1* sepal primordium was similar to that of the meristem by any parameter used (cell proliferation rate, changes in cell volume, heterogeneity in cell and clone volumes, growth isotropy) (Figures 1E, 1F, and 2A–2D). Similar results were obtained in three independent live imaging experiments (Figure S2).

We conclude that the cellular basis for tissue growth is different between the meristem and the early primordium. This change in growth regime depends on *JAG* and is necessary for proper primordium growth but is not required for primordium emergence.

### *JAG* Controls the Coordination between Cell Growth and Cell Cycle in Sepal Primordia

**Figure 3:**
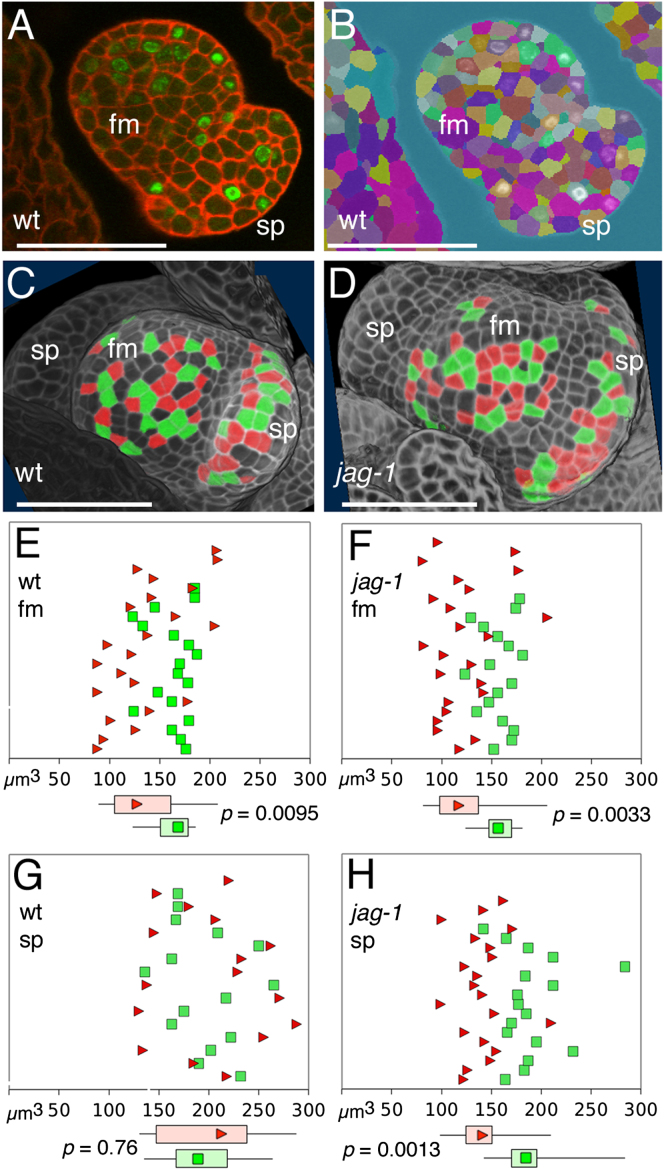
*JAG* Alters the Coordination between Cell Volume and S Phase Entry at the Transition from Meristem to Primordium Identity fm, floral meristem; sp, sepal primordium; scale bars represent 50 μm. (A) Single optical section through a WT bud at phyllotactic position 10, showing simultaneous imaging of cell walls (red; mPS-PI staining) and DNA synthesis (green; EdU incorporation). (B) Virtual section corresponding to (A), showing simultaneous EdU detection (white nuclei) and 3D segmentation; the segmented image was used to extract the volumes of individual cells (marked in different colors). (C) 3D projection of the complete stack of optical sections of the WT bud shown in (A), superimposed on 3D projections of selected segmented cells from (B) that showed EdU incorporation (green) and neighboring control cells with no EdU signal (red); the volumes of these cells were plotted in the graphs shown in (E) and (G). (D) 3D projection equivalent to that shown in (C) but for a *jag-1* bud at phyllotactic position 12; the volumes of EdU-positive cells (green) and control EdU-negative cells (red) were plotted in (F) and (H). (E–H) Scatterplots of the volumes of EdU-labeled cells (green squares) and control unlabeled neighboring cells (red triangles) for the WT floral meristem (E), *jag-1* floral meristem (F), WT primordium (G), and *jag-1* sepal primordium (H). The x axis shows cell volumes in μm^3^; the y axis does not represent any measurement and was used to spread out data points; the box plot below each graph indicates the minimum and maximum volumes (black line), volumes within the first to third quartiles (colored boxes), and the median volume (symbols inside the boxes). P values correspond to the null hypothesis that median cell volumes were the same for EdU-positive and -negative cells, tested using Wilcoxon’s signed rank test; p values for a replicate experiment (data not shown) were 0.0138 (E), 0.029 (F), 0.15 (G), and 0.010 (H).

Based on our observations that *JAG* promoted both cell proliferation and an increase in average cell volume, we next studied how cell cycle and cell volume are coordinated during meristem and primordium growth. Because it has been shown that inhibition of DNA synthesis stops meristem growth, whereas inhibition of M phase entry does not prevent meristem and primordium growth [16], we focused on the S phase as the growth-limiting step of the cell cycle. To monitor simultaneously cell volume and progression through S phase, we combined methods for high-resolution imaging of cell walls in deep tissues [17] with labeling of newly synthesized DNA using the nucleotide analog 5-ethynyl-2′-deoxyuridine (EdU) [18] (Figure 3A) and then applied the 3D segmentation step of MARS [2] to the cell wall images of flower buds (Figure 3B). By analyzing cells in the epidermal layer of the floral meristem and of the adaxial epidermis of sepal primordia, we were able to relate the data to the live imaging analysis shown in Figures 1 and 2.

In the floral meristem of both the WT and the *jag-1* mutant, cell volumes ranged from approximately 100 to 200 μm^3^ and EdU-labeled cells had a larger median volume than unlabeled cells (Figures 3E and 3F). This suggests that cell growth in the meristem occurs mostly before S phase and that DNA synthesis is initiated in cells that reach a threshold volume, as seen, for example, in budding yeast [19]. In accordance with the live imaging results, cell volumes in the WT primordium varied over a wider range of volumes (approximately 100–300 μm^3^) (Figure 3G). Different to the results in the meristem, EdU labeling did not correlate with cell volume in the primordium (Figure 3G). In contrast, the *jag-1* primordium once again was similar to the meristem, with a clear correlation between cell volume and DNA synthesis (Figure 3H). Consistent results were obtained for each genotype using buds at positions 10–12 in the phyllotactic spiral (data not shown); Figure 3 compares a WT bud at stage 10 with a *jag-1* bud at position 12 to compensate for the slower growth of *jag-1* primordia and ensure that the different cell behavior was not related simply to primordium size. In summary, cell volume and entry into S phase are coordinated in meristem cells and the transition to organ primordium identity included a *JAG*-dependent decoupling of S phase entry from cell volume.

### Ectopic JAG Decouples Cell Cycle from Cell Volume in the Meristem

The results above suggested that in primordia, *JAG* might override a mechanism that coordinates S phase entry and cell size or that *JAG* activates a mechanism that increases cell volume independently of the cell cycle, for example, by increasing vacuolar volume. Electron microscopy, however, indicated that vacuoles do not contribute significantly to cell volume in the adaxial epidermis of emerging sepal primordia (Figure 3). To test whether *JAG* is sufficient to decouple S phase entry from cell volume, and whether this is necessarily associated with an increase in cell volumes, we activated *JAG* ectopically in the floral meristem.

**Figure 4:**
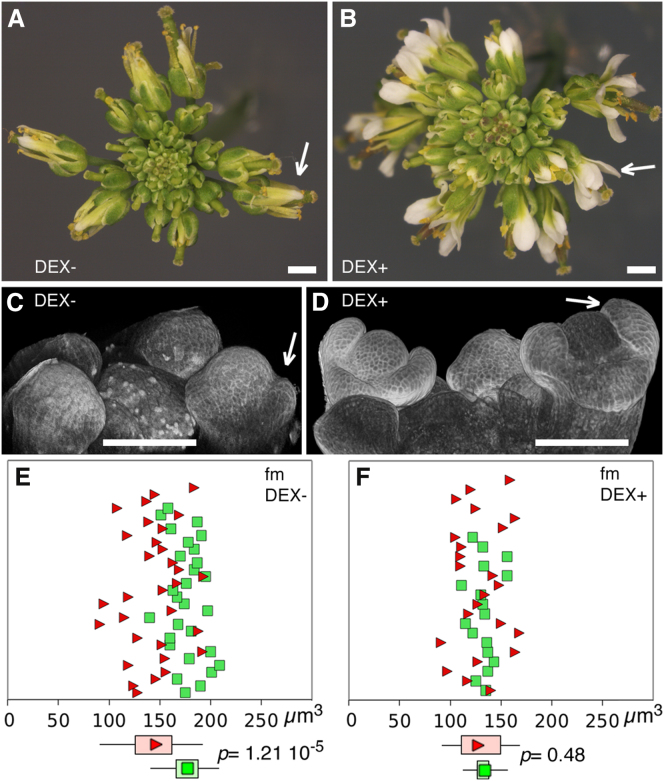
Ectopic JAG Activation Decouples S Phase from Cell Volume in the Floral Meristem (A–D) Rescue of floral organ development by JAG-GR; (A and B) *35S::JAG-GR, jag-2* inflorescence apices 2 weeks after mock treatment (A) or treatment with dexamethasone 10 μM (B). (C) and (D) show young buds of *35S::JAG-GR, jag-2* inflorescence apices grown for 3 days in medium without dexamethasone (C) or with dexamethasone 10 μM (D); arrows indicate rescue of organ growth after dexamethasone treatment; scale bars represent 1 mm (A and B) and 100 μm (C and D). (E and F) Scatterplots of the volumes of EdU-labeled cells (green squares) and control unlabeled neighboring cells (red triangles) in the floral meristem (fm) of buds at phyllotactic position 12 after 48 hr growth in medium without dexamethasone (E) or with 10 μM dexamethasone (F); imaging, image analysis and statistics were as described for Figure 4; p values (Wilcoxon’s signed rank test) for two additional replicate experiments (data not shown) were 1.28 10^−6^, 3.27 10^−3^ (E) and 0.60, 0.91 (F).

Sustained ectopic expression of JAG severely disrupts flower development [12]; therefore, we used transient activation of JAG in the floral meristem. For this, we generated plants (*35S::JAG:GR*) that expressed ubiquitously a fusion between JAG and the rat glucocorticoid receptor steroid-binding domain, which renders transcription factors dexamethasone-dependent [20]. Transient treatment of *35S:JAG-GR jag-2* inflorescences with 10 μM dexamethasone rescued organ growth at both early and late developmental stages, confirming that the JAG-GR fusion provided inducible *JAG* function (Figures 4A–4D). We then monitored cell volumes and S phase entry in the floral meristem of *35S::JAG-GR* buds at position 10–13 after incubation in media with or without 10 uM dexamethasone. Meristem cells of mock-treated buds showed again the coordination between cell volume and S phase entry described above (Figure 4E). After JAG-GR was activated, coordination between cell volume and S phase entry was lost in meristem cells, as seen before for WT sepal primordia (Figure 3). In contrast to the WT primordia, however, the median cell size in the meristem decreased in response to *JAG* activation, because cells entered S phase at abnormally small volumes (Figure 4F). Thus the *JAG*-induced decoupling of S phase entry from cell volume does not require a concomitant increase in median cell volumes. These results suggest that cells in the floral meristem have the potential to enter S phase at small volumes but are normally prevented from doing so by a mechanism that can be overridden by *JAG*.

### JAG Represses Meristem Development Genes

The results above revealed that cells in the *jag* mutant primordium behave like meristem cells in several ways: rate of growth, growth isotropy, cell enlargement, cell size homogeneity, and coordination between cell size and entry into S phase. This raised the question whether *JAG* antagonizes genes required for meristem development and whether these genes would be ectopically expressed in *jag* mutant primordia.

One of the key meristem regulators is *STM*, which encodes a KNOX-type homeodomain protein required for the establishment and maintenance of all shoot meristems [6]. *BP* is a close *STM* homolog that directs development of the stem and flower pedicels [7] but can also assume the role of *STM* in meristem maintenance [8]. Both the STM and BP proteins have been reported to function as heterodimers with TALE homeodomain proteins, of which BELL 1 (BEL1) is the best characterized, with roles in inflorescence meristem and ovule development [21, 22]. We therefore tested the regulatory effect of *JAG* on *STM*, *BP*, and *BEL1*. In the case of *STM*, repression by JAG-GR suggested that it might be a target of *JAG*, however, RNA in situ hybridization did not show changes in *STM* expression in the *jag-2* mutant (data not shown). In contrast, we saw that *BP* and *BEL1* were targeted both by ectopic and endogenous *JAG*, as detailed below.

**Figure 5:**
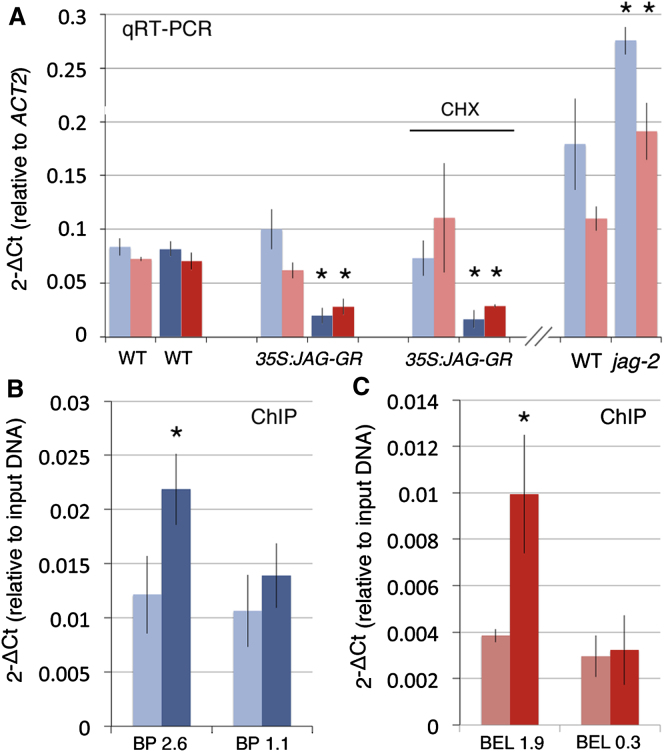
JAG Regulates *BP* and *BEL1* Error bars show the average and SD of three biological replicates; asterisks indicate statistically significant differences (unpaired two-sample Student’s t test, p < 0.05) between samples and corresponding controls. (A) Expression levels (relative to the *ACT2* constitutive control) of *BP* (blue) and *BEL1* (red) mRNA measured by qRT-PCR in inflorescence apices of WT (WT) or *35S::JAG-GR* plants 4 hr after mock treatment (pale colors) or treatment with dexamethasone 10 μM (dark colors). CHX indicates samples from plants that were also treated with cycloheximide 10 μM; expression in the WT and *jag-2* was compared in a separate experiment. (B and C) ChIP using anti-GR antibodies and inflorescence apices of *35S::JAG-GR* plants 4 hr after mock treatment (pale colors) or treatment with dexamethasone 10 μM (dark colors). (B) shows target sequences 2.6 or 1.1 Kb upstream of the *BP* start codon. (C) shows target sequences 1.9 or 0.3 Kb upstream of the *BEL1* start codon.

Both *BP* and *BEL1* were significantly downregulated 4 hr after activation of JAG-GR throughout inflorescence apices with dexamethasone (Figure 5A). Downregulation by JAG-GR still occurred in the presence of cycloheximide, showing that repression of *BP* and *BEL1* did not involve intermediate steps that required protein synthesis [23]. Chromatin immunoprecipitation (ChIP) was then used to test whether JAG-GR directly interacted with *BP* and *BEL1* in a dexamethasone-dependent way. In both cases, reproducible binding was seen to specific regions upstream of the starting codon (Figures 5B and 5C).

**Figure 6:**
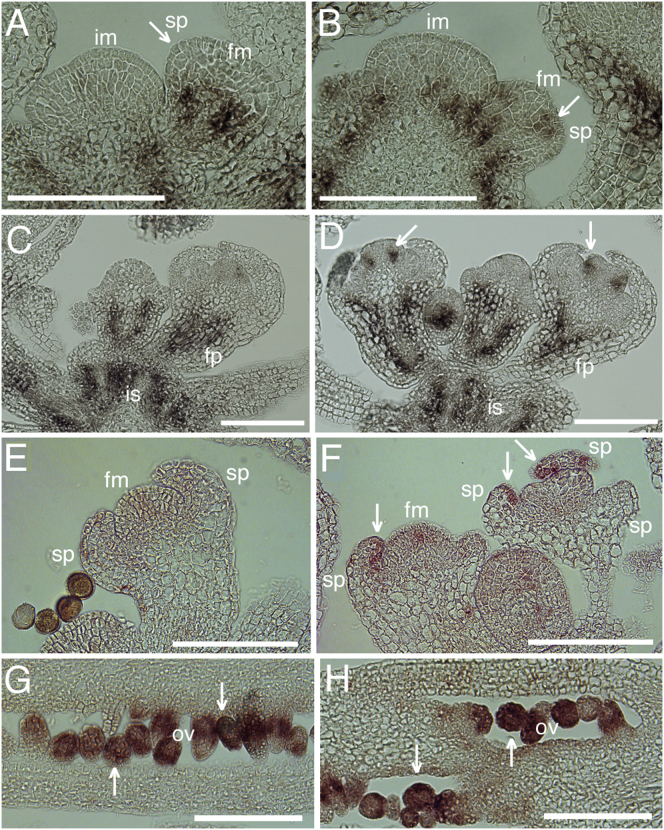
RNA In Situ Hybridization Showing that *BP* and *BEL1* Are Ectopically Expressed in *jag-2* fm, floral meristem; sp, sepal primordium; im, inflorescence meristem; is, inflorescence stem; fp, flower pedicel; ov, ovules; scale bars represent 100 μm. (A–D) Sections through WT (A and C) and *jag-2* (B and D) inflorescence apices hybridized with *BP* antisense probe; arrows indicate ectopic *BP* expression in sepal primordia (B) and in carpel primordia (D). (E and F) Sections through WT (E) and *jag-2* (F) floral buds hybridized with *BEL1* antisense probe; arrows indicate ectopic *BEL1* expression in sepal primordia. (G and H) Sections through WT (G) and *jag-2* (H) carpels hybridized with *BEL1* antisense probe; arrows indicate normal *BEL1* expression in developing ovules.

To verify that *BP* and *BEL1* are regulated by endogenous *JAG*, we compared expression in WT and *jag-2* young floral buds with RNA in situ hybridization (Figure 6). For *BP*, the known expression pattern in flower pedicels and stems [24] was comparable in WT and *jag-2* plants (Figures 6A–6D). In addition, *jag-2* buds showed ectopic expression at the base of organ primordia, both in emerging sepals (Figures 6A and 6B) and in the emerging carpel primordia of stage 6 buds [25] (Figures 6C and 6D). For *BEL1*, strong expression in ovules, which serve as a control for the known expression pattern, was similar in WT and in *jag-2* (Figures 6G and 6H). The low levels of *BEL1* expression reported in floral meristems [22] were not detected by in situ hybridization in our conditions, either in WT or *jag-2* buds (Figures 6E and 6F). In sections through the same inflorescence apices, however, clear expression of *BEL1* was seen in the sepal primordia of *jag-2* buds, but not in the WT controls (Figures 6E and 6F). qRT-PCR independently confirmed that the levels of *BP* and *BEL1* messenger RNA (mRNA) were increased in *jag-1* inflorescences apices relative to the WT (Figure 5A).

Ectopic *BP* expression causes leaf and floral defects reminiscent of those seen in the *jag* mutants [26], prompting us to test the functional consequences of ectopic *BP* expression in *jag*. The *jag-1 bp* double mutant, however, showed additive phenotypes, with the stem and pedicel defects seen in *bp* combined with the same organ development defects seen in *jag-1*, both at early and late stages (Figure S4). Therefore the organ growth defects seen in *jag* mutants are not caused primarily by ectopic *BP* expression.

We conclude that direct repression of *BP* and *BEL1* by *JAG* provides molecular evidence that *JAG* has a role in promoting the transition from meristem to primordium identity, in accordance with the changes in cellular parameters described above. However, the function of *JAG* in organ growth is likely to involve more than repression of meristem regulators.

## Discussion

The aim of this work was to understand how growth regulators direct the growth of organ primordia at the cellular level. Our analysis revealed that *JAG* is required for the transition from meristem to primordium growth patterns, including a shift from isotropic to anisotropic growth, increased cell proliferation and cell enlargement. Unexpectedly, we also observed a *JAG*-dependent loss of coordination between cell volume and DNA synthesis, associated with increased heterogeneity in cell sizes.

In accordance with a role for *JAG* in the transition from meristem to primordium identity, we show that *JAG* represses at least two meristem regulatory genes, *BP* and *BEL1*. It has been shown that the growth defects of *jag-2* sepals and petals are strongly enhanced by mutations in *ASYMMETRIC LEAVES 1* and *2* (*AS1* and *AS2*), which are known repressors of *BP* [27]. Combined with the genetic interaction between *jag-2* and *as1/2*, the direct interaction of JAG-GR with *BP* and *BEL1* and the ectopic expression of these genes in *jag-2* primordia reveal *JAG* as a novel member of the gene regulatory network that controls the transition from meristem to primordium identity.

In contrast to the regulatory network, the cellular basis for the transition from meristem to primordium identity has been less well characterized. Primordium emergence involves changes to the cell wall structure that facilitate cell expansion [28, 29]. Measurements of the geometry of surface cells have previously shown that the initiation of leaf primordia in Anagalis and floral buds in *Arabidopsis* is accompanied by increased growth rates and increased growth anisotropy [30, 31]. Analysis of the surface geometry of *Arabidopsis* sepal primordia at stages later than those reported here also revealed strong growth anisotropy and cell size heterogeneity [32]. However, it has been unclear to what extent the mechanisms involved in the emergence of primordia from the meristem are the same required for subsequent primordium growth and morphogenesis. We showed that in the *jag* mutant, sepal primordia still emerged and were physically distinguishable from the meristem, but their cells continued to grow in the same way as the meristem cells. Therefore, the changes in cell behavior that underpin primordium growth are genetically separable from those required for primordium emergence.

One of the consequences of *JAG* function in sepal primordia was increased cell size heterogeneity. In unicellular organisms, uniform cell sizes are maintained by cell size checkpoints [19, 33]. In multicellular organisms, the control of cell size homeostasis is much less well understood. It has been proposed that a cell size checkpoint operates to maintain a minimal cell size prior to mitosis to ensure daughter cell viability but that external, developmental inputs often override cell autonomous size checkpoints [19, 34, 35]. Our results suggest that a checkpoint for cell size operates during G1-S progression in the meristem and is deactivated in the primordium in a *JAG*-dependent way. Although we reveal *JAG* as an upstream regulator of cell size homeostasis during plant development, it remains to be seen whether JAG directly controls cell-autonomous mechanisms that couple cell growth and cell cycle or whether intermediate regulatory genes and signals are involved.

Regardless of the mechanism, there are several reasons why cell size and cell cycle may need to be coupled in the meristem, but not in sepal primordia. Cell sizes influence auxin transport through tissues [36], so irregularity of cell sizes could affect auxin-dependent meristem patterning. Conversely, increased variability in cell size may be required during differentiation in the primordium; the finding that cell size controls transcriptional responses [37] raises the possibility that changes in cell size may not only be a consequence but also a cause of differentiation. Additionally, a mechanism that triggered cell division at a threshold volume might impose a growth penalty that would be acceptable in the meristem but incompatible with the fast growth rates seen in organ primordia. Such a growth penalty could result from variation in growth rate during the cell cycle. For example, slower growth in M phase has been widely observed [38] and could be caused in plants by rearrangements of the cytoskeleton, which has a key role not only in mitosis but also in cell wall expansion [39]. Mathematical modeling will be required to explore these hypotheses further.

## Experimental Procedures

### Plant Material

*Arabidopsis thaliana* Landsberg-*erecta* (L-*er*) was used as the WT; *jag-1* [12], originally in Columbia (Col) background, was backcrossed three times into L-*er*; *jag-2* [13] was originally L-*er*. The *35S:JAG:GR* construct was generated in the binary vector pCGN1547 as described [40], transformed into L-*er* by floral dip [41] and selected for complementation after crossing to *jag-2*. For expression analysis and ChIP, plants were grown on soil in 16 hr light, 20°C/8 hr dark, 18°C cycles. For quantitative 3D imaging, plants were grown on soil at 20°C in short days (10 hr light/14 hr dark) for 4 weeks and subsequently transferred to 16°C, continuous light during flowering.

### Confocal Microscopy

For time-lapse imaging, inflorescence apices were prepared and imaged as described [2]. For combined mPS-PI (modified pseudo-Schiff-propidium iodide) [17] and EdU [18] imaging, inflorescence tips were dissected and buds larger than 0.5 mm were removed. The dissected apices were grown for 45 hr in sterile GM medium [42] at 16°C under continuous light; for JAG-GR activation, the media also contained 10 μM dexamethasone (from 10 mM stock in ethanol) or 0.1% ethanol for mock treatment. Apices were then transferred to the same medium supplemented with 10 μM EdU (Invitrogen) and grown for another 3 hr, followed by 15 min each in 15%, 30%, 50%, 70%, 85%, 95%, and 100% ethanol. After 16 hr in ethanol and further dissection leaving only the inflorescence meristem and buds up to position 15–16, the samples were rehydrated through the same ethanol series and incubated at 37°C overnight in alpha-amylase (Sigma) 0.3 mg/mL in phosphate buffer 20 mM pH7.0, 2 mM NaCl, 0.25 mM CaCl_2_. All subsequent steps were at room temperature with gentle shaking: first, the apices were rinsed in water and incubated for 1 hr in solution containing 10 μM Alexa 488-azide (Invitrogen) and 100 mM Tris pH 8.5; this was followed by 30 min in solution contining 10 μM Alexa 488-azide, 100 mM Tris, 1 mM CuSO_4_, 100 mM ascorbic acid, pH 8.5; the apices were subsequently washed three times in water, treated 30 min in 1% periodic acid, washed twice in water, and incubated 2 hr in Schiff-PI reagent [17]. The samples were finally cleared with chloral hydrate solution and mounted in Hoyer’s medium [17], before imaging with a Zeiss 510 Meta confocal microscope with excitation at 488 nm and emission filters set to 572–625 nm for propidium iodide and 505–600 nm for EdU.

### Image Processing and Analysis

Image alignment and segmentation was performed with mars-alt version 1 (http://openalea.gforge.inria.fr/doc/vplants/vtissue/doc/_build/html/user/mars_alt_v1/index.html) [2]. For segmentation of mPS-PI images, the EMPILER and SEGMENTATION scripts of mars_alt v1 were used; zviewer and zfuse (mars_alt v1) were used to overlap segmented images with EdU images and to mark selected cells. A custom Python script was used to edit segmented images and retain only selected cells. Segmented images from MARS were imported into Matlab (MathWorks) and cell volumes were calculated based on the number of voxels per cell using custom Matlab scripts. Measurement of growth isotropy in epidermal sectors was based on growth tensors as described [3], using projections of the sectors onto a plane minimizing the sum of squared distances. Statistical analysis was performed with RCommander (http://socserv.mcmaster.ca/jfox/Misc/Rcmdr/). Custom scripts are available upon request.

For image display, MARS image files (.inr.gz) were imported into ImageJ64 (http://rsbweb.nih.gov/ij/download.html) using LOCI (http://loci.wisc.edu/bio-formats/imagej) and displayed in 3D using the 3D Viewer plugin (http://rsb.info.nih.gov/ij/plugins/) (Figures 1, 3C, 3D, and 4); projections of virtual sectors were also created with 3D viewer and cell clones were tracked manually and colored with Adobe Photoshop CS4 (Adobe Systems). Figure 3A was created with the ImageJ 3D Viewer plugin and Figure 3B with zfuse (mars-alt v1). Photoshop CS4 was used for final editing of the images (cropping, sizing, brightness and contrast).

### Quantitative RT-PCR

For JAG-GR activation, inflorescences were dipped once into 0.015% Silwet L-77 (De Sangosse) 0.1% ethanol solution and supplemented with dexamethasone and cycloheximide as described in Figure 5. After 4 hr in daylight, inflorescence apices (only unopened flower buds) of 12 plants were collected per sample in three biological replicates per treatment. RNA was extracted using the RNEasy plant mini kit (QIAGEN), treated with Ambion® DNA-*free* (Invitrogen) and reverse transcribed using oligo (dT) 12–18 (Invitrogen), Superscript III reverse transcriptase (Invitrogen) and RNasin RNase Inhibitor (Promega) according to the manufacturers’ instructions. Quantitative PCR was performed in technical triplicates using primers BEL1-F, BEL1-R, BP-F, BP-R, STM-F, and STM-R (Supplemental Information) with the LightCycler 480 System and SYBR Green I (Roche). Data were normalized to *ACTIN*2 (amplified with primers ACT2-F, ACT2-R; Supplemental Information) as described [43].

### Chromatin Immunoprecipitation

JAG-GR was activated as described above and ChIP was as described [44], except for the following modifications: 70–80 inflorescence apices were used per sample; fixation buffer was modified to pH 8.5 and phenylmethylsulfonyl fluoride (PMSF) used at 0.1 mM; after grinding in liquid nitrogen, 300–500 mg tissue powder was resuspended in 700 μl of lysis buffer, modified to contain 0.1 mM PMSF and two tablets of protease inhibitor cocktail complete Mini, EDTA-free (Roche) added per 50 ml of buffer; the supernatant after sonication was precleared for 2 hr with 25 μl Dynabeads Protein A beads (Invitrogen) equilibrated in lysis buffer with 1 mg/ml BSA and 20 μg/ml sonicated salmon sperm, then incubated with 2 μl GR-antibodies (AB3580, Abcam) per 100 μl lysate at 4°C, overnight; 15 μl of equilibrated Dynabeads Protein A beads were added per 100 μl lysate and incubated at 4°C for 4 hr, before proceeding with washes and decrosslinking as described [44]. Q-PCR was as described above using primers BEL3947-F/BEL3947-R, BELUTR-F/BELUTR, BP2609-F/BP2609-R, and BP1064-F/BP1064-R (Supplemental Information).

### RNA In Situ Hybridization

*BEL-1* complementary DNA (cDNA) (nt 442–2077) and *BP* cDNA (full length) were cloned in *pBluescript KS(–)*. Probes were transcribed in vitro from linearized plasmids using the DIG RNA Labeling kit (Roche) and T7 RNA Polymerase (Roche). In situ hybridization was performed as described [14] and imaged using a Leica DM 6000 microscope.

## Accession Numbers

Sequence data can be found in the *Arabidopsis* Genome Initiative database under the accession numbers *JAG* (AT1G68480), *BP* (AT4G08150), *BEL-1*(AT5G41410), and *ACT2* (AT3G18780).

## Supplemental Information

Document S1. Figures S1–S4, Table S1, and Supplemental Experimental Procedures
