# Supplementary material for: JAGGED Controls Growth Anisotropy and Coordination between Cell Size and Cell Cycle during Plant Organogenesis
Source: Curr Biol. 2012 Oct 9;22(19):1739–46. doi: 10.1016/j.cub.2012.07.020 (PMC3471073; doi:10.1016/j.cub.2012.07.020)
Supplement: Document S1. Figures S1–S4, Table S1, and Supplemental Experimental Procedures [file mmc1.pdf]

Current Biology, Volume *22*

Supplemental Information

JAGGED Controls Growth Anisotropy and Coordination between Cell Size and Cell Cycle during Plant Organogenesis

Katharina Schiessl, Swathi Kausika, Paul Southam, Max Bush, and Robert Sablowski

Supplemental Inventory

1. Supplemental Figures and Tables

Figure S1, related to Figure 1

Figure S2, related to Figure 2

Figure S3, related to Figure 3

Figure S4, related to Figure 6

Table S1

2. Supplemental Experimental Procedures

3. Supplemental References

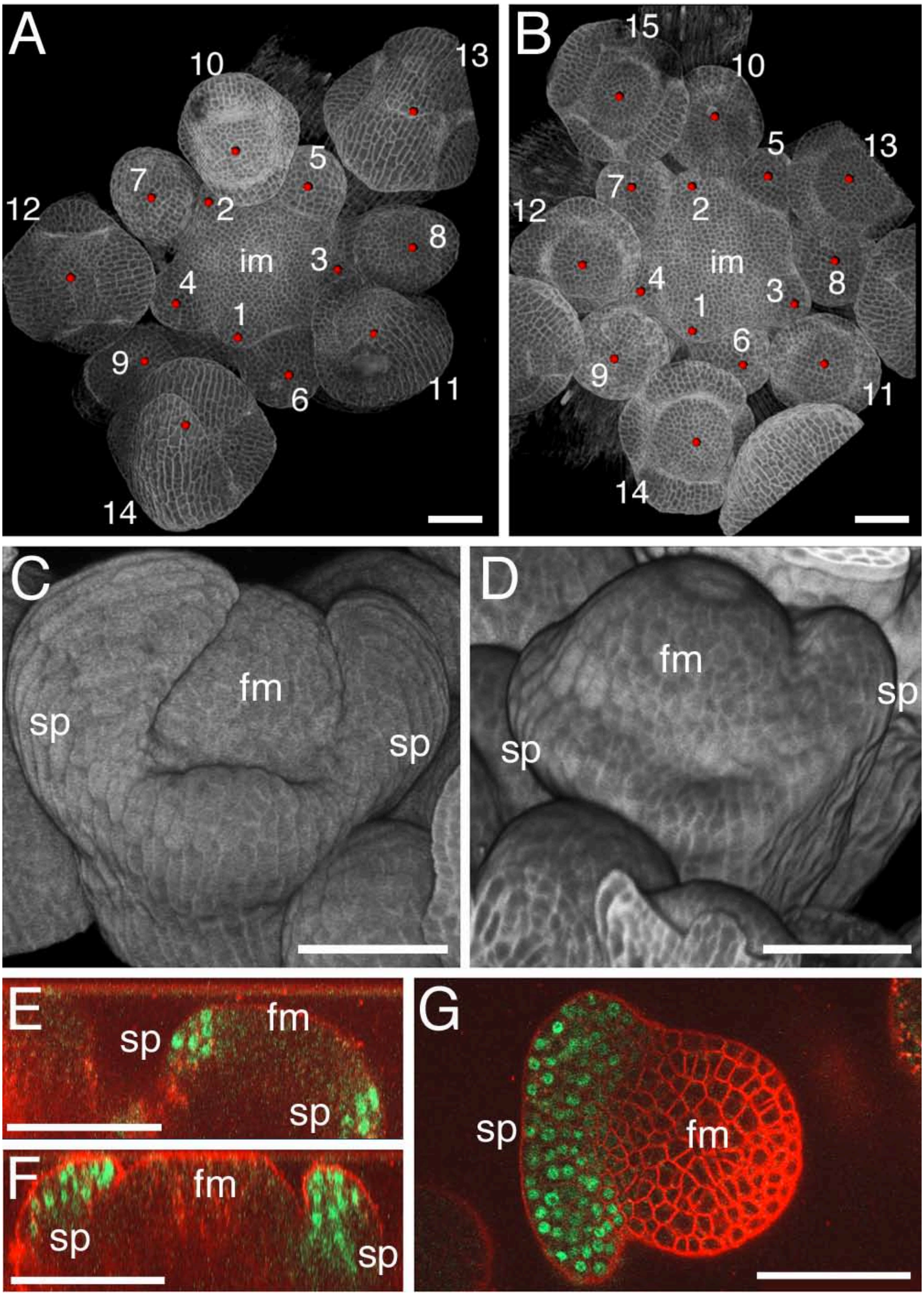

A
10
13
7
5
12
2
im
3
8
4
1
9
6
11
14
B
15
10
5
13
7
2
12
im
4
3
8
1
6
9
11
14
C
fm
sp
sp
D
fm
sp
sp
E
sp
fm
sp
F
fm
sp
sp
G
sp
fm

Figure S1. *jag-1* Defects in Early Flower Development, Related to Figure 1

Inflorescence meristem: im; floral meristem: fm; sepal primordium: sp; scale bar: 50 µm.

(A,B) Top view of wild-type (A) or *jag-1* (B) inflorescence apices (3D reconstruction from optical sections of mPS-PI stained apices); numbers indicate the phyllotactic position of buds around the inflorescence meristem, starting with 1 for the earliest visible floral bud and increasing with bud age.

(C,D) 3D Reconstructions of wild-type (C) and *jag-1* (D) floral buds at phyllotactic position 12; growth of the sepal primordia flanking the floral meristem is inhibited in *jag-1*, while the floral meristem was unaffected (maximum meristem width at the base of sepal primordia was 88.7 ± 6.7 µm for the wild type and 85.7 ± 2.9 µm for *jag-1*; mean ± SD, n=3).

(E-G) Expression of *pJAG:JAG-GFP* in wild-type sepal primordia, seen in medial optical sections through floral buds at phyllotactic positions 9 (E) and 11 (F) and in a section through the base of a sepal primordium of a bud at position 13 (G).

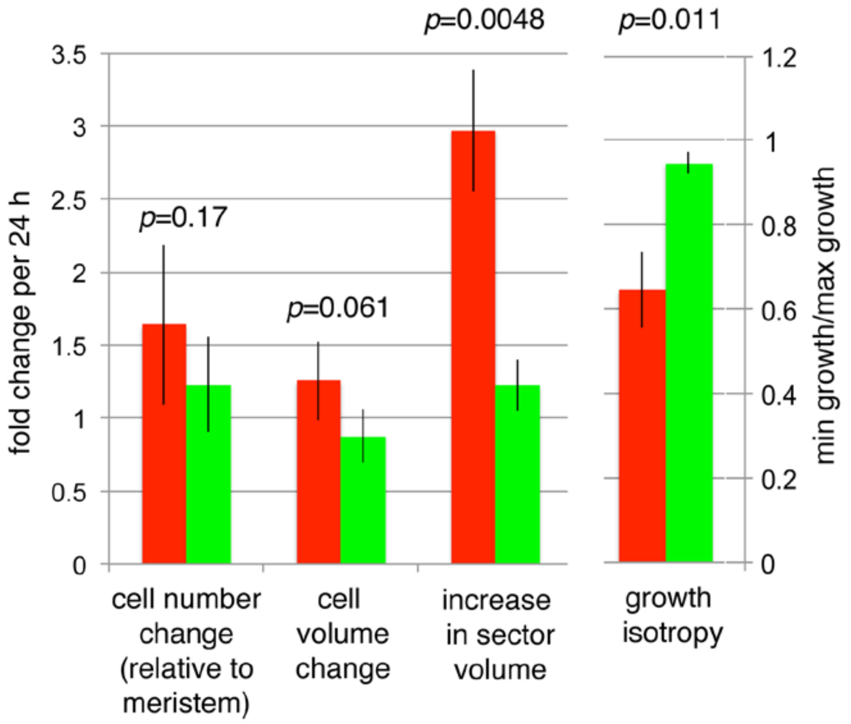

Figure S2. Summary Statistics of Independent Live Imaging Experiments Comparing Growth of Wild-Type and *jag* Sepal Primordia, Related to Figure 2

Bars represent average and standard deviation of growth parameters of sepal primordia in three experiments; *p*-values correspond to the null hypothesis that wt and *jag* had the same average (Student's *t*-test, n=3). The single value used for each experiment was the average value for the growth parameter across the time course. Values for cell number, cell volume and sector volume correspond to fold change per 24 h interval; the rate of cell proliferation in the sepal primordia is expressed relative to the proliferation rate in the meristem of the same flower. Growth isotropy is the ratio between growth rates along the axes of minimum and maximum growth over a 24 h interval. Wt (red) was always Landsberg-*erecta* (L-*er*); *jag* (green) was *jag-1* (backcrossed to L-*er*) in one experiment and *jag-2* (originally in L-*er*) in two other experiments.

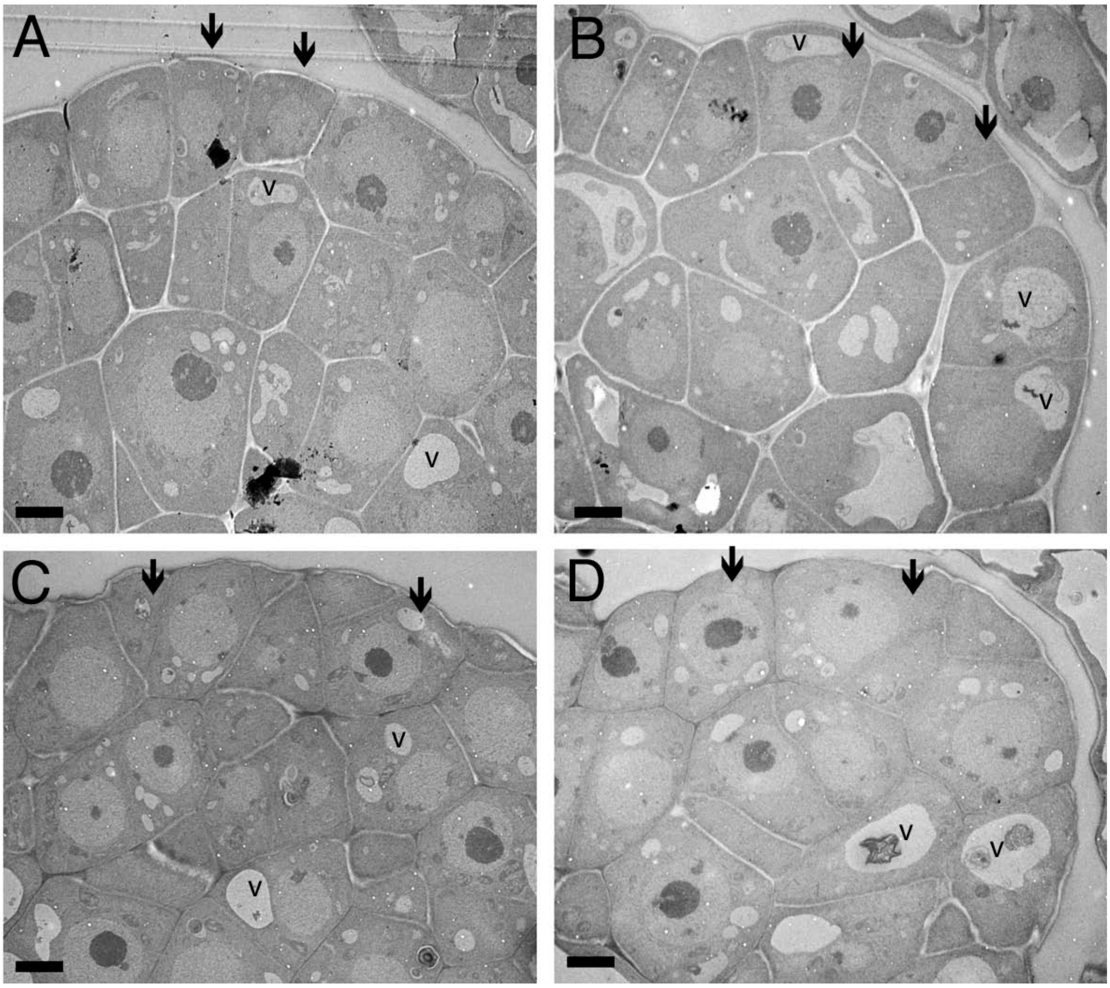

Figure S3. Transmission Electron Micrographs of Sections through Floral Buds at Stages Corresponding to Phyllotactic Positions 10-12, Related to Figure 3

Vacuoles: v; arrows indicate epidermal cells in positions comparable to those selected for quantitative analysis (Figures 2,3,4); scale bar: 2 µm.

(A) wild-type floral meristem

(B) wild-type sepal primordium

(C) *jag-1* floral meristem

(D*) jag-1* sepal primordium

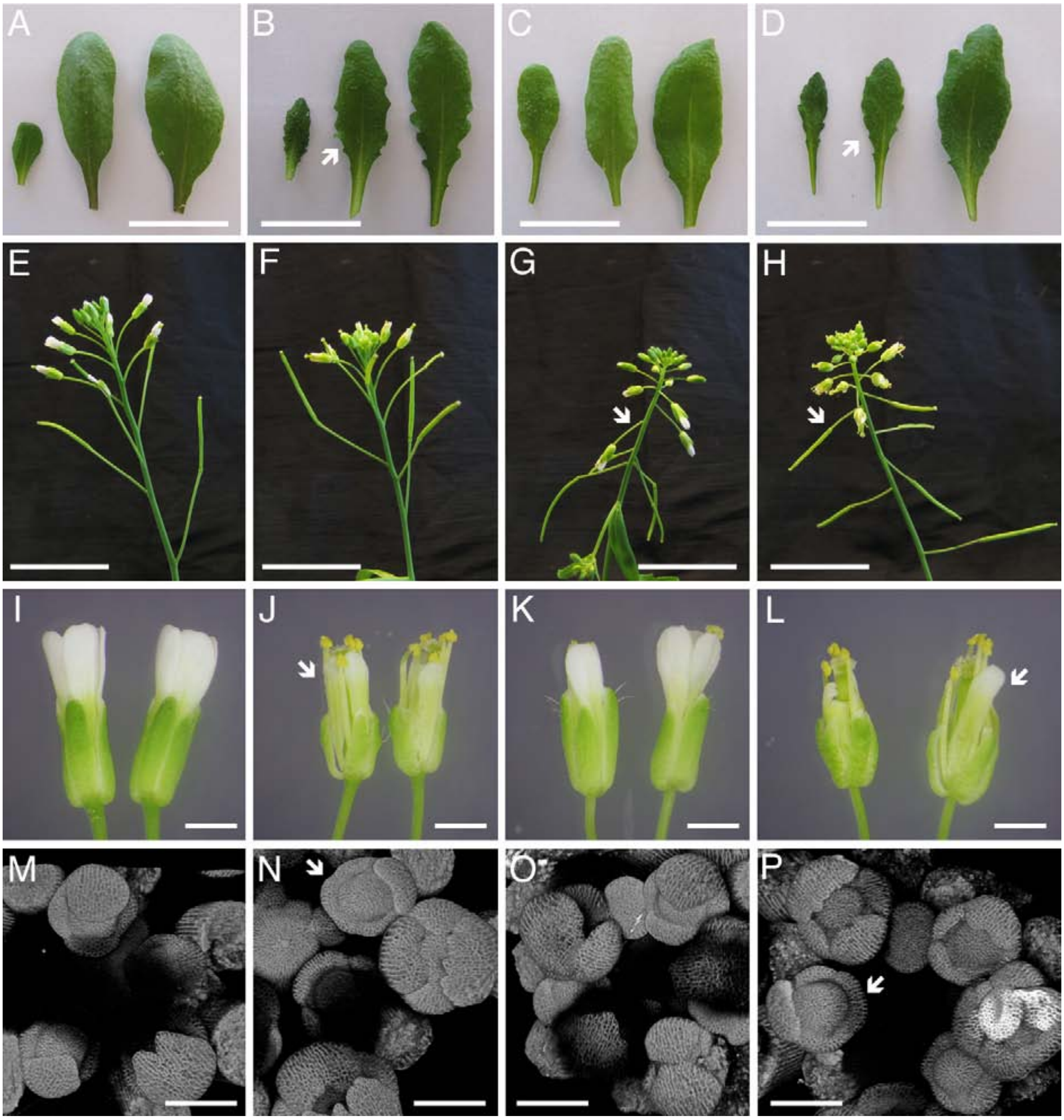

Figure S4. Additive Phenotypes of the *jag* and *bp* Mutations, Related to Figure 6

Scale bars: 1 cm (A-H); 1 mm (E-H), 100 µm (I-L).

(A,E,I,M) Wild-type (Col); (B,F,J,N) *jag-1*; (C,G,K,O) bp; (D,H,L,P) *jag-1 bp*.

(A-D) Rosette leaves; arrows indicate serrates leaves in *jag-1* and *jag-1 bp*.

(E-H) Inflorescence apices; arrows indicate the downward-turned siliques typical of *bp*.

(I-L) Close-up of mature flowers; arrows indicate the defective perianth growth characteristic of *jag* mutants.

(M-P) 3-D reconstruction of confocal images of young floral buds; arrows point at buds with the defective sepal primordia characteristic of *jag* mutants.

Table 1. Oligonucleotide Sequences

ACT2-F	ATGGAAGCTGCTGGAATCCAC
ACT2-R	TTGCTCATACGGTCAGCGATG
BEL3947-F	TCTGAGAAAAGCCTGAACTC
BEL3947-R	GTTGGTGTATAGATGGAGAGGT
BP2609-F	GGCAGTGCAATGAAGTGAAA
BP2609-R	CCCGAAACATAAAACCTACACG
BP1064-F	GCAAGACTTGGATGTTTTTGG
BP1064-R	TCGGATAGTGTGATCTCTCCAC
BELUTR-F	CTTCTCTCTCCCTCTTCACC
BELUTR-R	AGCTCCTAAAACCCTACAAGA

Supplemental Experimental Procedures

Plant Material:

To produce *pJAG::JAG-GFP*, PCR was used to generate a 9.7 kb *JAG* genomic fragment (chromosome location 25682925 – 25692696) with the stop codon replaced by an *Nco*I site, where the sGFP S65T coding sequence [1] was inserted in frame before cloning in the binary vector *pPZP222* [2]. Transgenic lines were generated by floral dip transformation [3] of L-*er* and selected for segregation as single loci and for complementation after crossing to *jag-2*. To produce *jag bp* double mutants, *jag-1* (in the original Col background) was crossed to a *bp* allele isolated in Col (gift from M. Byrne, University of Sydney; the allele has a deletion of the *bp* promoter and shows the typical phenotype seen in severe *bp* loss-of-function alleles; [4]). Double mutants were selected phenotypically in the F2 and confirmed by PCR genotyping.

Confocal Imaging

For Supplementary Figure 1 A-D, mPS-PI (modified pseudo-Schiff-propidium iodide) was performed as described)[5]. For supplementary Figure 1 E-G and Supplementary Figure 4 I-L, dissected inflorescence apices were imbibed for 10 min in 50 µg/ml N-(4-triethylammoniumpropyl)-4-(p-dietheylaminophenylhexatrienyl pyridium dibromide)(FM4-64, Invitrogen) and imaged with a Zeiss 510 Meta confocal microscope with excitation at 488 nm and emission filters set to 572-625 nm for FM4-64 and 505-600 nm for GFP. Osirix (http://www.osirix-viewer.com/) was used for 3D reconstructions of mPS-PI images (Supplementary Figure 1 A-D). Meristem measurements were performed with ImageJ64 (http://rsbweb.nih.gov/ij/download.html). Virtual sections through pJAG::JAG-GFP images (Supplementary Figure 1E-G) and the 3D reconstructions of young buds (Supplementary Figure 4 I-L) were created with the Volume Viewer and 3D Viewer plugins of ImageJ64 (http://rsb.info.nih.gov/ij/plugins/). Photoshop CS4 (Adobe Inc.) was used for final editing of the images (cropping, sizing, brightness and contrast).

Electron Microscopy

Inflorescence apices were fixed in 2.5% GA/0.05M Na cacodylate, pH 7.2, vacuum infiltrated, left overnight, post-fixed in 1% osmium tetroxide/0.05M Na cacodylate for 1 hour, washed with water and dehydrated up to 1hr each step in ethanol 30%, 50%, 70%, 90% and 100%. Samples were then infiltrated in LR white resin and sectioned for TEM imaging with a FEI Technai G2 20 Twin TEM.

Supplemental References

1. Chiu, W., Niwa, Y., Zeng, W., Hirano, T., Kobayashi, H., and Sheen, J. (1996). Engineered GFP as a vital reporter in plants. Curr Biol *6*, 325-330.
2. Hajdukiewicz, P., Svab, Z., and Maliga, P. (1994). The small, versatile pPZP family of Agrobacterium binary vectors for plant transformation. Plant Mol.Biol. *25*, 989-994.
3. Clough, S.J., and Bent, A.F. (1998). Floral dip: a simplified method for Agrobacterium-mediated transformation of Arabidopsis thaliana. Plant J. *16*, 735-743.
4. Venglat, S.P., Dumonceaux, T., Rozwadowski, K., Parnell, L., Babic, V., Keller, W., Martienssen, R., Selvaraj, G., and Datla, R. (2002). The homeobox gene BREVIPEDICELLUS is a key regulator of inflorescence architecture in Arabidopsis. Proc Natl Acad Sci U S A *99*, 4730-4735.
5. Truernit, E., Bauby, H., Dubreucq, B., Grandjean, O., Runions, J., Barthelemy, J., and Palauqui, J.-C. (2008). High-resolution whole-mount imaging of three-dimensional tissue organization and gene expression enables the study of phloem development and structure in Arabidopsis. The Plant Cell *20*, 1494-1503.
